# The epidemiology of invasive fungal infections in transplant recipients

**DOI:** 10.1016/j.bj.2024.100719

**Published:** 2024-04-04

**Authors:** Dorra Elhaj Mahmoud, Anaïs Hérivaux, Florent Morio, Benoit Briard, Cécile Vigneau, Guillaume Desoubeaux, Jean-Philippe Bouchara, Jean-Pierre Gangneux, Gilles Nevez, Solène Le Gal, Nicolas Papon

**Affiliations:** a University of Angers, University of Brest, Infections Respiratoires Fongiques, SFR Interactions Cellulaires et Applications Thérapeutiques, Angers, France; b Nantes Université, CHU Nantes, Cibles et Médicaments des Infections et de L'Immunité, UR1155, Nantes, France; c INSERM, Centre d'Etude des Pathologies Respiratoires (CEPR), UMR 1100, Université de Tours, Faculté de Médecine de Tours, Tours, France; d CHRU Tours, Parasitologie-Mycologie Médicale-Médecine Tropicale, Tours, France; e Laboratory of Parasitology and Mycology, Brest University Hospital, Brest, France; f University of Brest, University of Angers, Infections Respiratoires Fongiques, SFR Interactions Cellulaires et Applications Thérapeutiques, Brest, France; g University of Rennes, CHU Rennes, Inserm, EHESP, Irset (Institut de Recherche en Santé, Environnement et Travail), UMR_S, 1085, Rennes, France; h Laboratory of Parasitology and Medical Mycology, European Confederation of Medical Mycology (ECMM) Excellence Center, Centre National de Référence Aspergilloses Chroniques, Rennes University Hospital, Rennes, France; i Division of Nephrology, Rennes University Hospital, Rennes, France

## Abstract

Transplant patients, including solid-organ transplant (SOT) and hematopoietic stem cell transplant (HSCT) recipients, are exposed to various types of complications, particularly rejection. To prevent these outcomes, transplant recipients commonly receive long-term immunosuppressive regimens that in turn make them more susceptible to a wide array of infectious diseases, notably those caused by opportunistic pathogens. Among these, invasive fungal infections (IFIs) remain a major cause of mortality and morbidity in both SOT and HSCT recipients. Despite the continuing improvement in early diagnostics and treatments of IFIs, the management of these infections in transplant patients is still complicated. Here, we provide an overview concerning the most recent trends in the epidemiology of IFIs in SOT and HSCT recipients by describing the prominent yeast and mold species involved, the timing of post-transplant IFIs and the risk factors associated with their occurrence in these particularly weak populations. We also give special emphasis into basic research advances in the field that recently suggested a role of the global and long-term prophylactic regimen in orchestrating various biological disturbances in the organism and conditioning the emergence of the most adapted fungal strains to the particular physiological profiles of transplant patients.

## Highlights

• Transplant patients are exposed to invasive fungal infections (IFIs).
• The management of these infections in transplant patients is still complicated.
• We provide an overview of the epidemiology of IFIs in transplant patients.
• Long-term prophylactic regimen may influence emergence IFIs in transplant patients.

## 1 Introduction

Fungal infections, also referred to as mycoses, represent a global burden worldwide. While superficial fungal infections account for a large proportion of the overall prevalence of mycoses and are fortunately rarely fatal, invasive fungal infections (IFIs) are characterized by dramatic high morbidity, mortality, and economic burden. Indeed, IFIs kill approximately 1.5 million people annually, a mortality rate three times greater than that of malaria, influenza, or breast cancer [1]. IFIs most often affect critically ill patients and those with significant underlying immune system disorders. Populations at greatest risk of IFIs include patients with hematological malignancies, critically ill patients in intensive care units [2], and transplant recipients [3].

The ever-increasing number of patients undergoing transplantation procedures is evidenced by the worldwide activity of more than 200,000 grafts per year, including solid organ (SOT) or hematopoietic stem cell (HSCT) transplantations [4]. Transplant patients are then exposed to various types of complications such as rejection and infectious diseases, of which IFIs are among the most important [[5], [6], [7]]. IFIs are mainly caused by *Candida* spp., *Aspergillus* spp., and to a lesser extent, by *Cryptococcus* spp., Mucorales, *Pneumocystis jirovecii* and other filamentous fungi. Proven IFIs are defined as the presence of fungal elements in tissues by biopsy or needle aspirates after histological and/or cultural investigations. IFIs are considered probable if the fungus is identified from bronchoalveolar lavage fluid or sputum when consistent clinical features and host factors are present. Possible IFIs include cases with appropriate host factors and with sufficient clinical evidence but lacking mycological support [5,7,8].

Despite improvements in immunosuppressive regimens and prophylactic strategies, IFIs still remain a major cause of morbidity and mortality in both SOT and HSCT recipients [[5], [6], [7]].

The present review aims to detail the current epidemiological landscape of IFIs after SOT and HSCT and highlight the recent data on their main risk factors. We also discuss recent findings suggesting that prophylaxis regimens may condition the onset of IFIs in transplant recipients.

## 2 Overview of the epidemiology of IFIs in SOT and HSCT

**Fig. 1:**
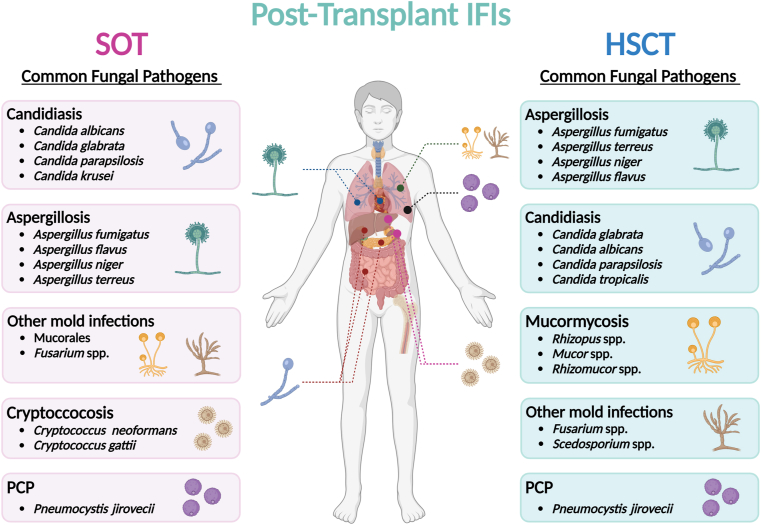
**Epidemiology of invasive fungal infections (IFIs) in solid organ transplant (SOT) and hematopoietic stem cell transplant (HSCT) recipients.** Pathogens are represented based on their prevalence in SOT and HSCT. Invasive candidiasis are the most common IFIs in SOT recipients, followed by invasive aspergillosis, non-*Aspergillus* molds infections, cryptococcosis and finally *Pneumocystis jirovecii* pneumonia (PCP). Liver, small bowel and pancreas transplant recipients have the highest rates of *Candida* infection. The highest incidence of aspergillosis is observed among lung and heart transplant recipients. Non-*Aspergillus* mold infections and PCP are mainly observed in lung transplants. Kidney and liver transplant recipients have the highest rates of cryptococcosis. Invasive aspergillosis are the most common IFIs in HSCT recipients, followed by invasive candidiasis, mucormycosis, other mold infections and finally PCP.

IFIs represent a major challenge in patients who underwent SOT and HSCT [3,7]. Based on American and European surveillance networks in adult and pediatric transplant recipients, the 12-month incidence of IFIs ranges from 1.3 to 11.6% in SOT patients and 3.4–3.7% in HSCT patients, with a changing epidemiology over time [[9], [10], [11]]. Indeed, while *Candida albicans* and *Aspergillus* spp. remain the most important pathogens in SOT and HSCT, a rising emergence of non-*C. albicans* and non-*Aspergillus* infection has recently been noted [12]. The Transplant-associated infection Surveillance Network (TRANSNET), a global repository of data on transplant patients followed-up prospectively over a six-year period (from 2001 to 2006), found that the risk of IFIs in SOT varied with the organ type, being highest in small bowel (11.6%), followed by lung (8.6%), liver (4.7%), heart (4%), pancreas (3.4%), and kidney (1.3%) [5]. The knowledge of these epidemiological differences is important to implement appropriate strategies for IFI prevention. In the HSCT population, the overall incidence of IFIs was approximately 8% in unrelated or mismatched allogeneic HSCT, 6% in matched related allogeneic HSCT, whereas a low incidence (less than 2%) was observed in autologous HSCT [6,11] [Fig. 1].

### 2.1 Trends in fungal infections in the SOT population

Although better outcomes have been reported with the use of targeted antifungal prophylaxis in selected high-risk recipients, IFIs are being increasingly recognized in SOT recipients [[13], [14], [15]].

Candidiasis are the most common IFIs among SOT types, with the exception of lung transplant recipients in whom *Aspergillus* infections are more prevalent [5,9]. In non-pulmonary SOT recipients, *Candida* infections account for 49%–85% of all IFIs [16]. Invasive candidiasis are most frequently observed in small bowel, pancreas, and liver transplant recipients, as many *Candida* species are natural commensals of the human gastrointestinal tract [5,17]. In the SOT population, *C. albicans* is the dominant pathogen, but an epidemiological trend toward non-*albicans* species has been observed in some studies [[17], [18], [19], [20]]. *Candida glabrata* (now renamed *Nakaseomyces glabratus*) is the most common of the non-*C. albicans* etiologic agent*,* accounting for approximately 40% of the total *Candida* isolates. *C. glabrata* infections are predominantly observed in liver and kidney-liver transplant recipients. *Candida parapsilosis* (∼6%) and *Candida krusei* (now renamed *Pichia kudriavzevii*) (∼5%) are also important pathogens in SOT recipients, especially in those who have received prior antifungal therapy [21,22]. Recently, *Candida auris* infections have been reported to cause outbreaks in liver and kidney transplant recipients [20,23]. Overall, invasive candidiasis remains associated with high rates of morbidity, mortality, and excess healthcare costs [24]. Mortality at 12 weeks after diagnosis ranges from 20 to 40% and appears to be particularly high for non-*C. albicans* infections [24,25].

Although *Candida* spp. remain the most common cause of IFIs in SOT recipients, molds account for approximately a quarter of IFIs in this population [9,24]. As expected, due to their ubiquitous presence in the environment, *Aspergillus* species represent the majority of them [5,14]. The incidence of invasive aspergillosis in SOT appears to be highly variable depending on the transplanted organ, ranging from 8.6% for lung and heart-lung, to 4.7%, 4.0%, and 3.4% for liver, pancreas and kidney-pancreas, and heart transplant recipients, respectively, whereas only 1.3% of kidney transplant recipients in the same study experienced an invasive pulmonary aspergillosis [5]. *Aspergillus fumigatus* is by far the most frequently isolated species regardless of the type of organ transplant [14,26]. Infections caused by *Aspergillus flavus*, *Aspergillus niger* and *Aspergillus terreus* have also been reported but are less common (10.2%, 10.2% and 3.1% respectively) [14,22,27,28], except in some tropical areas, likely due to the elevated prevalence of these species in the environment. Invasive aspergillosis remains associated with high graft loss and mortality in the SOT population. Indeed, the overall mortality rate at 12 weeks after diagnosis is as high as 15–25% in non-liver SOT recipients and up to 80–90% in liver SOT recipients, particularly in those undergoing retransplantation after 30 days of primary transplant. In liver transplant recipients, invasive aspergillosis presents more frequently as a disseminated disease than in other transplant types, except for heart [14,22,27,29].

Other molds, such as Mucorales, *Scedosporium/Lomentospora* spp., *Fusarium* spp., *Scopulariopsis/Microascus*, *Paecilomyces* spp. have been described as emerging causes of IFIs [30,31]. However, many other fungi may complicate the management of SOT recipients, including dimorphic fungi [32,33]. The primary mode of acquiring mold infections is inhalation of fungal airborne spores. The resulting IFIs are predominantly observed in lung transplant recipients and are associated with higher mortality due to the frequent multiresistance of these fungi [12].

Cryptococcosis is the third most common fungal infection in SOT recipients, accounting for approximately 8% of IFIs in this population [34]. The overall incidence of cryptococcosis in SOT recipients ranges from 0.2% to 5% [34,35]. The majority of cases occur in kidney and, to a lesser extent, in liver transplant recipients [5,35] and are mainly caused by *Cryptococcus neoformans* and *Cryptococcus gattii* stricto sensu [36]. Mortality rates for invasive cryptococcosis typically range from 33% to 42% in the SOT population. Importantly, infections caused by *C. gattii* have been shown to have a mortality rate of up to 70% in SOT recipients, despite an early initiation of an appropriate therapy [36,37].

The remaining infections are due to many other fungi, particularly *P. jirovecii* [[38], [39], [40]]. The epidemiology of *P. jirovecii* in non-human immunodeficiency virus (HIV) patients has evolved significantly over the past two decades, and its incidence has substantially increased in SOT recipients [40,41], while its incidence has decreased in HIV-infected patients due to HIV detection improvement, antiretroviral therapy and PCP prophylaxis [7].The trend of increasing PCP among SOT recipients has been especially highlighted by the French surveillance network of invasive fungal infections (RESSIF), with an incidence of 15.2% [7]. According to Cheng and colleagues in 2022 [40], lung transplant recipients have the highest risk of *P. jirovecii* pneumonia (PCP) (5.7%), compared with kidney (1.4%) and liver (0.7%) transplant recipients. A previous report from the United Kingdom (UK) patients estimated the incidence of PCP among SOT recipients to be 5.8%, 5.5%, 1.2%, and 0.3% for lung/heart and lung, heart, liver, and kidney transplantations, respectively [42]. This is probably due to the more intense immunosuppressive therapy given to the lung/heart and lung transplant recipients [39]. A French multicenter retrospective analysis reported a PCP incidence of July 2, 1000 per year in lung transplant patients [43]. A dramatic increase in PCP incidence has been reported in kidney recipients. In the UK, an approximately 4-fold increase (38.8%) in the number of PCP cases among kidney recipients was observed from 2006 to 2010, while the number of renal transplantations increased by only 25% [44]. These observations are in accordance with those from a recent French nation-wide survey [45], which showed an increasing number of PCP outbreaks with common genotype strain among transplant recipients, especially among kidney transplant recipients. Increased number of PCP cases may be related to greater frequency of immunosuppressant applications, to increased person-to-person transmission (spread in the health care environment), or to improvements in diagnostic methods [44].

### 2.2 Trends in fungal infections in the HSCT population

IFIs are one of the major limiting factors for the successful outcome of patients receiving HSCT, especially in allogeneic HSCT [46]. In older series, *Candida* spp. were the primary etiology of IFIs in the HSCT population with *C. albicans* being the most prevalent species [47]. At that time, *Aspergillus* spp. were diagnosed in less than 6% of HSCT recipients, but the mortality rate was nearly 100% [48].

In the 1990s, a revolution in antifungal prophylaxis changed the epidemiological landscape of IFIs in the HSCT population, with a marked increase in the incidence of molds, especially *Aspergillus* spp [48,49]. According to a consistent number of recent studies evaluating the epidemiology of IFIs in the HSCT population [6,11], invasive aspergillosis is the most prevalent, accounting for 43–64% of the infections. As in the SOT population, *A. fumigatus* is the most frequently isolated *Aspergillus* species in HSCT recipients, followed by *A. terreus*, *A. niger* and *A. flavus* [3]. A variability in the incidence of invasive aspergillosis is also observed in these patients depending on the transplanted cell type, ranging from 0.4 to 6.7% [6] and time to transplantation. The overall 1-year mortality in cohorts with invasive aspergillosis is dramatic, reaching 70% in some studies [6,48,50].

The epidemiology of invasive mold infections continues to evolve at an alarming rate. Indeed, the emergence of non-*Aspergillus* molds, such as Mucorales, *Fusarium* spp. and *Scedosporium* spp. has been noted [51]. According to the TRANSNET and the Prospective Antifungal Therapy (PATH) databases, mucormycosis accounts for 7–8% of IFIs in the HSCT population [6,52], and the SEIFEM study reported *Fusarium* infections in 0.11% of HSCT cases [11]. These results differ sharply from those reported in Brazil, where a prevalence of 5.2% for *Fusarium* infections was found in allogeneic HSCT recipients, higher than those observed for candidiasis (2.4%) and aspergillosis (2.3%) [53]. Although poorly documented, it's crucial to recognize that the ecological characteristics, and consequently the local fungal biota, of the various countries where transplantation occurs likely influence the epidemiology of IFIs in high-risk patients. This factor may account for the variations in incidence observed across different studies. According to TRANSNET data, the 1-year survival in the HSCT cohort was lowest in patients with *Fusarium* infections (6.3%) [6]. In a retrospective case series of 61 patients with an invasive *Scedosporium/Lomentospora* infection, including 17 HSCT recipients, the overall mortality was 70% [54].

Despite the significant decrease in their incidence, *Candida* species remain the second most common causative agents of IFIs in the HSCT population after *Aspergillu*s spp. Non-*albicans Candida* species account for almost 70% of all *Candida* infections in the TRANSNET study [6]. In this study, *C. glabrata* (33%) ranked first followed by *C. albicans* (20%). In addition, *C. parapsilosis* (14%), *Candida tropicalis* (8%), and *C. krusei* (6%) emerged as important pathogens in the HSCT population. The 1-year survival rate in patients with candidiasis was 33.6%, slightly higher than in other IFIs [6].

In contrast to SOT recipients, the epidemiology of cryptococcosis in HSCT recipients is poorly documented. Currently, only a few cases of cryptococcosis have been reported in HSCT recipients, with *C. neoformans* being the main species (up to 61.9% of the causative species) [[55], [56], [57]]. PCP has also been described in HSCT recipients although its incidence is low due to highly effective prophylaxis regimens. Analysis of the data from the Center for International Blood and Marrow Transplant Research (CIBMTR) registry between 1995 and 2005 revealed that 0.63% of allogeneic recipients and 0.28% of autologous recipients of a first HSCT developed PCP [58]. A recent retrospective analysis by Coda and colleagues [59] reported nine cases of PCP among 2082 patients undergoing autologous HSCT, for an incidence of 0.43%.

## 3 Timing of post-transplant IFIs

**Fig. 2:**
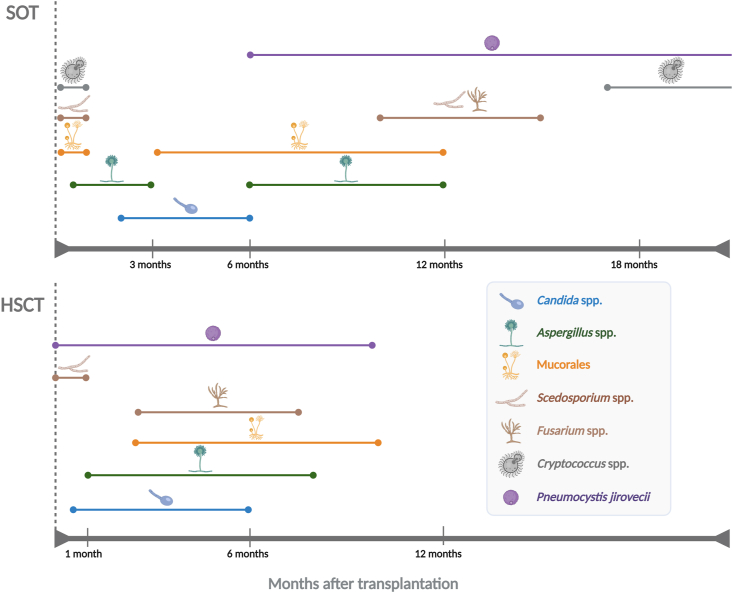
Timing of invasive fungal infections (IFIs) after solid organ (SOT) and hematopoietic stem cell (HSCT) transplantations.

Time is a determining factor in post-transplant IFIs and influences the type of infections that transplant patients may develop. Many factors influence the occurrence of IFIs after SOT and HSCT, including the type of transplant, the use and duration of antifungal prophylaxis, and the degree of immunosuppression. In general, in the early post-transplant period, the etiologic cause of IFIs is often found in pre-existing donor-recipient pathogens and nosocomial infections. Late post-transplant IFIs are mainly caused by opportunistic pathogens and reactivation of latent infections [3,60] [Fig. 2].

### 3.1 Timing of IFIs following SOT

The timing of IFIs after SOT has been investigated in several prospective studies [5,60,61]. In the TRANSNET report, the majority of IFIs onsets occurred relatively late, typically more than 3 months after transplantation [5].

In general, invasive candidiasis is the earliest complication of SOT [24]. With current antifungal prophylaxis strategies, the time to onset of invasive candidiasis ranges from 2 to 6 months [5,22,25]. In heart transplant recipients, invasive candidiasis occurs rapidly in the first 100 days after transplantation, possibly related to the frequent use of catheters in this setting [22].

The median time to onset of invasive aspergillosis is 184 days [5]. However, the onset of aspergillosis is closely related to the site of infection. For instance, tracheobronchial or anastomotic *Aspergillus* infections typically occur within 90 days, whereas other forms of invasive aspergillosis occur later, between 6 and 12 months after SOT [5,22,62,63] [Fig. 2].

Mucormycosis and other non-*Aspergillus* mold infections in SOT patients have been reported in several studies in the late post-transplant period, with a mean time to onset of 10–15 months [5]. However, more recent data suggest that mucormycosis tends to occur earlier after transplantation. In liver recipients, it may occur as early as the first month after transplantation, while in other SOT it may occur 3–6 months or later [64]. For *Scedosporium* spp. and *Fusarium* spp., infections occur within the first 12 months after lung, kidney, and liver transplantation [65]. Nevertheless, it is important to remember that the timeline of non-*Aspergillus* mold infections is largely dependent on the prior colonization of the respiratory tract in SOT recipients. For example, scedosporiosis can develop as early as one month after transplantation in those with a pre-transplant airway colonization [66]. Random amplification of polymorphic DNA from clinical isolates recovered from previously colonized patients with cystic fibrosis who underwent a fatal IFI following lung or heart/lung transplantation, revealed identical genotypes to those colonizing the airways up to two years before transplantation, demonstrating that prior airway colonization by non-*Aspergillus* molds is an important risk factor for IFIs [67,68].

Cryptococcosis is one of the latest infectious complications following SOT [34,36]. Infection usually occurs after *de novo* inhalation of the fungus (yeast form or basidiospores), from an environmental source, or after reactivation of a latent infection [69,70]. The median time to onset of cryptococcosis is approximately 1.5 years after transplantation, but varies by transplanted organ [36]. Time to onset is generally earlier in both liver and lung transplant recipients compared to kidney transplant recipients [36], and in rare cases, donor-derived cryptococcosis has been described to occur in the recipient within 30 days of transplantation [36,71]. In this regard, a high attention is required for organs provided from donors with unexplained neurological illness or meningoencephalitis [36].

PCP, which is an increasing problem in SOT patients, usually corresponds to nosocomial infections. Since *P. jirovecii* circulates in human populations through airborne interindividual transmission, sometimes causing clonal outbreaks in the hospital environment, PCP generally develops at any time after SOT and has an incubation period which can vary from 3 weeks to 4.5 months [72,73]. A recent study reported that SOT recipients have an increased risk of PCP at 6 months, 1 year, 2 years, and 3 years after transplantation [40].

### 3.2 Timing of IFIs following HSCT

In the HSCT population, the timeline of IFIs is divided into three distinct post-transplant periods: early onset (≤1 month [pre-engraftment phase]), late onset (1–6 months [post-engraftment phase]), and very late onset (>6 months) [61]. In the TRANSNET cohort, the median time to IFIs after HSCT is 61 days for candidiasis, 99 days for aspergillosis, 123 days for fusariosis, and 135 days for mucormycosis [6]. The PATH registry reported similar results, with a median time from HSCT to IFIs of 83 days for invasive aspergillosis and 108 days for invasive candidiasis [52]. However, a difference in the onset of invasive candidiasis is however observed between autologous and allogeneic HSCT. Indeed, invasive candidiasis tends to occur earlier after autologous HSCT (median 28 days) compared to allogeneic HSCT (median 108 days) [52]. *Scedosporium* infections typically occur in the first 30 days after transplantation and are more common in patients with multiple transplant procedures [74]. For cryptococcosis, the time to onset varies widely among HSCT patients and the infection may occur a few days after stem cell transplant administration to 5 years after transplantation [57,75]. PCP develops both early (between day 0–60) and late (beyond day 270) after HSCT. Approximately 50% of the cases occur between day 60 and 270 after autologous and allogeneic transplantation [58].

## 4 Risk factors for IFIs in SOT and HSCT

**Fig. 3:**
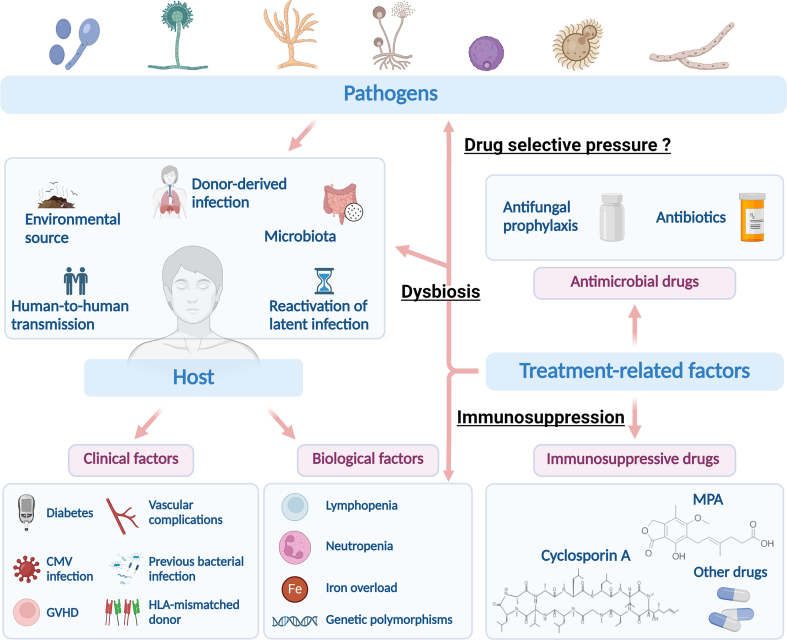
**A schematic overview of the factors influencing the pathogenesis of invasive fungal infections in transplant recipients.** Primary fungal infections following transplantation may be due to microorganism inhalation from an environmental source such as soil or hospital environment (e.g. Mucorales, *Cryptococcus* spp., *Aspergillus* spp. *Pneumocystis jirovecii*), a previously quiescent infection reactivation (e.g. *Cryptococcus* spp.), infected donor organs and tissue grafts (e.g. *Cryptococcus* spp., *Scedosporium apiospermum*) or from an endogenous source with the patient's flora (e.g. *Candida* spp.). Several clinical and biological parameters have been described as risk factors for IFIs after SOT and HSCT. Predisposing factors include fungal virulence traits and host characteristics. Another major determinant factor is the use of immunosuppressive and antimicrobial drugs. These molecules interfere with the patient's flora and antifungal immune response which impact the type of IFI and its time of onset. GVHD: Graft-vs-host-disease; HLA: Human leukocyte antigen; CMV: Cytomegalovirus; MPA: mycophenolic acid.

The susceptibility of SOT and HSCT recipients to IFIs appears to be multifactorial [2]. Indeed, the etiologic pathogens can be predicted based on the epidemiologic exposures of both the recipient and the donor, the patient's state of immunosuppression, and the immunological defect [61,76]. These effects are often exacerbated by treatment-related factors, including immunosuppressive medications and antimicrobial prophylaxis [2] [Fig. 3].

### 4.1 Prominent risk factors for IFIs in SOT

Many risk factors for IFIs after SOT have been identified. A meta-analysis including studies published between 2010 and 2019 identified several risk factors associated with IFIs after liver transplantation [77].The most relevant independent risk factors are vascular complications, especially hepatic vascular thrombosis and renal failure. Retransplantation, reoperation, model for end-stage liver disease (MELD) score ≥30, biliary leaks, and living donor liver transplantation are also frequently associated with IFIs in liver transplantation [78,79]. In kidney transplant recipients, diabetes, bacterial pneumonia, and urinary tract infections have been reported as major clinical risk factors for IFIs [80]. In lung transplant recipients, in which IFIs are frequent [81], previous fungal colonization constitutes a major risk factor for early-onset invasive mold infections [82]. Single-lung transplantation represents one of the major risk factors for invasive aspergillosis after lung transplantation. *Aspergillus* infections in single-lung transplant recipients likely represent reactivation of a preexisting focus, suggesting that the native lung may serve as a fungal nidus. Furthermore, patients who undergo single-lung transplantation have a higher prevalence of chronic obstructive pulmonary disease, a condition that may predispose to airway colonization by *Aspergillus* [62,83]. Other factors leading to late-onset invasive aspergillosis in lung recipients include age, immunosuppression, and bronchiolitis obliterans syndrome have also been described [81].

The association between specific infections and risk factors has been highlighted in the literature. For instance, in liver transplantation, previous bacterial infection and previous antibiotic use are considered as risk factors for invasive candidiasis. Post-transplant renal replacement therapy, reoperation, and cytomegalovirus (CMV) infection have been associated with invasive aspergillosis [77]. Previous studies [39,84] on liver transplant recipients described lymphopenia, CMV-related disease, steroid pulse therapy, recurrent hepatocellular carcinoma, and age at liver transplantation greater than 65 years as risk factors for PCP. Immunomodulating infections (CMV, tuberculosis and hepatitis C), longer duration of high-dose steroid therapy and mycophenolate acid -based regimen have been associated with an increased incidence of PCP in kidney transplant recipients [39].

Several host genetic polymorphisms have been associated with an increased susceptibility to IFIs after SOT. In this regard, a study exploring the role of host genetics in IFIs susceptibility showed that functional polymorphisms in *IL1B* and *DEFB1* (encoding human β-defensin 1) are associated with invasive mold infection in SOT recipients [85]. The Swiss Transplant Cohort Study described specific genetic polymorphisms in the gene encoding pentraxin 3 (*PTX3*) as risk factors for invasive mold infection in SOT recipients [86]. These polymorphisms are responsible for reduced immunity to molds, especially *Aspergillus*, as PTX3 can directly bind to *Aspergillus* conidia and activate complement and subsequent phagocytosis [86,87].

### 4.2 Prominent risk factors for IFIs in HSCT

Several risk factors for IFIs have been described in HSCT recipients. They encompass clinical risk factors (e.g., Human leukocyte antigen (HLA)-mismatched donors, severe chronic graft-versus-host disease (GVHD), diabetes, malnutrition, and CMV reactivation) and biological factors (e.g., iron overload, persistent neutropenia, multiple cell line deficiency, and genetic risk factors) [51,88]. Over a five-year period from 1998 to 2002, independent risk factors for invasive mold infections were analyzed in 1248 patients undergoing allogeneic HSCT [51]. The early emergence of invasive mold infections (<40 days after HSCT) is influenced by underlying disease and transplant-related factors, such as unrelated/mismatched HSCT and biological risk factors, including hyperglycemia and iron overload. Since iron is an essential element for fungal growth, elevated serum ferritin has been associated with a higher risk of developing invasive fungal infections in HSCT recipients. Lymphopenia has also recently been described as a risk factor for early invasive mold infections, especially aspergillosis, as *Aspergillus*-specific CD4^+^ T cell responses are crucial for pulmonary mold defense and may exhibit antifungal effector activity [89,90]. Various HSCT complications, including CMV disease, high transfusion frequency and severe acute GVHD, are associated with late invasive mold infections (40–100 days after HSCT) [51]. The RISK study by Choi and colleagues [91] identified the presence of underlying pulmonary disease and prolonged neutropenia (≥3 weeks) as additional risk factors for early IFIs after allogeneic HSCT. In the late phase, high ferritin levels, use of secondary immunosuppressive agents for refractory GVHD, and CMV reactivation are associated with IFIs. In the very late phase (101–365 days after HSCT), risk factors for IFIs include secondary neutropenia, severe chronic GVHD, and the use of a TNF-α inhibitor for refractory GVHD [91]. It is striking that despite similar clinical and biological risk factors, some patients appear to be more prone to develop IFIs after HSCT than others.

As in SOT recipients, polymorphisms in genes involved in immune responses against fungal pathogens constitute a major risk for IFIs after HSCT. Two non-synonymous polymorphisms in *Toll-like receptor 4* (*TLR4*) (D299G and T399I) in HSCT donors are considered as risk factors for invasive aspergillosis [92,93]. A stop codon polymorphism in *C-type lectin domain containing 7A* (*CLEC7A*) (encoding Dectin-1) in both recipients and donors is associated with invasive aspergillosis after HSCT, supporting the important role of Dectin-1 in the immune response against *Aspergillus* infection [94]. A study by Granell and colleagues [95] showed that polymorphisms responsible for mannose-binding lectin (MBL) and MBL-associated serine protease (MASP-2) deficiency are independent predictive factors for IFIs after allogeneic HSCT. A haplotypic variant in PTX3 is also associated with invasive aspergillosis in HSCT recipients [96]. All these findings highlight the importance of pre-transplant genetic analysis in the prediction and monitoring of IFIs after HSCT [97].

## 5 Concluding remarks and perspectives

Over the past two decades, we have observed marked changes in the epidemiology of IFIs in transplant recipients. These shifts may be primarily related to the continuous increase in the number of transplantation procedures that substantially increments annually the total cohort of individuals living with SOT or HSCT, and thus at high risk for IFIs. Improvements in diagnostic tools and the evolution of transplant practices may also have influenced these epidemiological changes. Although it is still too early to be sure, it is likely that climate change is gradually participating in these epidemiological modifications by modulating the local fungal flora in the environment of transplant recipients and transplantation units.

Although it is likely to be highly patient-dependent, and notably associated with individual predisposing factors, we now have a global overview of the timing of the onset of the most prominent fungal pathogens. Fortunately, this has considerably participated over the past decade in anticipating, preventing and improving the management of IFIs in both SOT and HCST patients.

Above all, recent advances in this field teach us that the global chemoprophylactic regimen must be considered as one of the predisposing factors that condition the onset of IFIs in SOT and HSCT populations. While possible drug interactions impacting PK/PD and toxicity, but also adverse effects of the distinct drugs used may be deleterious factors for transplantation outcome, this issue remains largely unexplored. Historically, most of the pioneering research focused on the increase of antifungal resistance and its impact on the management of transplant recipients, particularly for *Candida* spp. when the use of antifungal prophylaxis with fluconazole became recurrent [98,99]. Over the years, drug tolerance to echinocandins has also been observed and deeply investigated [100]. For instance, *C. glabrata*, which is the most frequently isolated non-*C. albicans* species in SOT and HSCT, may show a decrease in the susceptibility to some antifungal classes. This feature likely confers a selective advantage to this pathogen in the case of triazole and echinocandin prophylaxis [101].

The global influence of the remaining part of the prophylactic regimen, *i.e.* antibacterial and immunosuppressive drugs, on the fungal populations hosted by transplant patients (e.g., intestinal mycobiota, skin microflora, pulmonary colonization, …) and the origin of fungal strains subsequently responsible for invasive disease has long been an understudied field. In recent years, some groundbreaking studies have been published, providing unprecedented insight into the sequential events orchestrated by the antimicrobial regimen in transplant patients and its role as a predisposing factor for IFIs. In fact, while the gastrointestinal source of *Candida* spp. strains recovered from blood samples of HSCT recipients has long been the most obvious hypothesis, its experimental proof has been lacking. The first report by Zhai and colleagues [102] in 2020, attempted to decipher the origin of candidemia in HSCT. Taking advantage of new high-resolution sequencing approaches on fecal samples and bloodstream isolates, they demonstrated that: *i*) the antibacterial prophylaxis regimen first leads to lower levels of total bacterial burden and diversity in the gut of transplant patients; *ii*) this gut microbiota dysbiosis then conditions a global intestinal fungal burden, mainly composed of *Candida* spp.; *iii*) the prophylactic antifungal regimen (here based on micafungin) drives the emergence of less susceptible *Candida* isolates to echinocandins with severe pathogenic attributes (*C. parapsilosis* complex); and *iv*) these *Candida* isolates finally undergo random translocation to the blood vessels, leading to candidemia [103]. This advance was further confirmed by Rolling and colleagues [104] in 2021 using a similar approach in a larger cohort of HSCT patients.

In addition to prophylactic antimicrobials, immunosuppressants must also be considered for their potential in conditioning IFIs in transplant patients [105]. Indeed, due to their natural antifungal activity or their unexpected potential to modify fungal metabolism, immunosuppressants may promote the selection of initial genotypes and possibly induce the emergence of acquired resistance. This hypothesis of a potential selective effect of immunosuppressants on *A. fumigatus*, *C. neoformans*, and *C. albicans* causing IFIs in transplant patients dates back two decades ago but could not be confirmed [[106], [107], [108]]. However, recent advances have shown that mycophenolic acid (MPA), an immunosuppressive natural product used in the post-transplant maintenance protocol, may be involved in the selection of specific *P. jirovecii* genotypes [45,109] [Fig. 3]. As an example, a large cohort of patients diagnosed with PCP and treated or not with MPA were retrospectively examined by our team. The acquired data showed that MPA treatment is associated with a unique alanine to threonine substitution at position 261 (A261T) in the *P. jirovecii impdh* gene. This mutation has already been related to MPA resistance in other fungi [109]. Thus, these recent studies serve as a proof of concept that an immunosuppressant can promote the selection and emergence of specific fungal strains in transplant patients. As a consequence, these findings justify integrative scientific programs aiming at exploring the *in vitro* and *in vivo* influence of long-term exposure of prominent opportunistic fungal pathogens to currently used immunosuppressants.
